# A rare case of Cushing’s disease concurrent with papillary thyroid carcinoma

**DOI:** 10.22088/cjim.12.4.618

**Published:** 2021

**Authors:** Zahra Kashi, Omid Emadian, Marzieh Movahedirad

**Affiliations:** 1 Diabetes Research Center, Mazandaran University of Medical Sciences, Sari, Iran; 2 Department of Pathology, Mazandaran University of Medical Sciences, Sari, Iran

**Keywords:** Cushing’s disease, Papillary thyroid carcinoma

## Abstract

**Background:**

Although a nodular thyroid disease is higher in patients with pituitary adenoma, concurrent thyroid cancer with pituitary tumor is uncommon.

**Case Presentation:**

We report a young woman with discovered papillary thyroid carcinoma after 1-year transsphenoidal surgery for Cushing’s disease. Thyroidectomy was done and patient is well after three years follow-up.

**Conclusion:**

We suggest the patient with functional pituitary adenoma be evaluated for thyroid tumor.

Nodular thyroid disease (NTD) is one of the common thyroid disease and is frequent in general population. The most important risk factors are iodine deficiency, goitrogenic foods ,radiations ,aging ,genetics, gender and smoking (1).An increased frequency of NTD has also been reported with functional pituitary adenomas especially with acromegaly and after that is more associated with prolactinoma and Cushing’s disease (2). However, concomitant papillary thyroid carcinoma and pituitary adenomas are not common, especially Cushing’s disease. 

## Case Presentation

A 33-year-old woman with a history of depression and anti-depressant drugs therapy was referred to the endocrine clinic due to headache, weight gain about 16 kilograms and purple stria on the skin during last year. The patient's medications included sertraline and quetiapine, which weight gain continued despite quetiapine discontinuation and change to bupropion. The menstrual cycle was normal but the patient complained of hypomenorrhea and occasional galactorrhea since last year. She did not mention history of hirsutism, hypertension and diabetes mellitus. On physical examination, blood pressure was 120/80 mmhg and weight was 101 kg. Given the mentioned symptoms, the patient was evaluated for Cushing’s syndrome. In primary laboratory findings, morning basal plasma cortisol was 29 mic/dl and UFC (urinary free cortisol) 82.7 mic/dl (in normal range of laboratory). Morning fasting cortisol was not suppressed after 1 mg dexamethasone suppression test (9.9 mic/dl). Liddle test (low dose dexamethasone suppression test) was done and the results for serum and urinary cortisol were 10 micgr/dl and 15.5 micgr/dl respectively. The ACTH level was 118 pg/ml and a 14*15 mm pituitary adenoma was found in MRI. The patient’s visual field was normal. Except for prolactin which was high (907 miu/ml: normal up to 495), other pituitary axis functions were normal (table 1). 

**Table 1:** Pre pituitary surgery laboratory finding

**Laboratory Test**	**Result**	**Normal range:**
TSH (mIU/L)	1.6	0.5-5.5
FreeT4 (ng/dl)	0.8	0.8-1.7
Prolactin (Miu/l)	907	132-498
IGF1(ng/ml)	289	107-246
GH base(ng/ml)	0. 9	0.06-6
GH 2 hours after 75 gr glucose (ng/ml)	0.2	<1
NA (meq/l)	136	135-145
K (meq/l)	4	3.5-5.2
WBC (10^3^/micl)	13900 (PMN 75%, lym 25%)	4000-11000
FBS (mg/dl)	95	70-99
Anti Tpo (unit/ml)	1.3	<30

The patient was treated with cabergoline 0.5mg/week for 3 months, though the prolactin level decreased to 10 miu/ml but the pituitary adenoma did not shrink and finally she underwent TSS (Trans spheroidal surgery) with the diagnosis of corticotroph adenoma. In a pathology report, pituitary tumor was confirmed. In IHC study, the GH and ACTH markers were positive in majority of the tumor cells, but TSH, FSH, LH, P53 and prolactin were negative. Synaptophysin and chromogranin A were positive and ki67 was positive in 1% of tumoral cells. 

After pituitary surgery prednisolone tablet, 5 mg/d and nasal desmopressin spray was started and is continued until now. The weight gain stopped and the skin steria disappeared. After 1 year follow-up, the patients was well but complained of lumpy filling in neck. The thyroid examination was suspicious for thyroid nodule in right lobe. Morphologic characteristic of the nodule in sonography included a solid hypoecho signal, 10*9 mm in size, ill defined, taller than wide without calcification in right thyroid lobe. In addition, another 6*4 mm nodule with benign appearance was reported in right thyroid lobe. A sonography guided fine needle thyroid nodule aspiration (FNA) was performed. The thyroid cytopathologic diagnosis was atypia (or follicular lesion) of undetermined significance (AUS/FLUS). The FNA of thyroid nodule was repeated 3 months later and the papillary thyroid carcinoma diagnosis was reported in second cytology report. Total thyroidectomy was performed. In final pathology report, the tumor size was 1 cm without extrathyroidal extension and capsular or vascular invasion and there was one follicular adenoma in right lobe. No lymph node was involved. Based upon the clinicopathologic report and low risk of recurrence (papillary thyroid cancer confined to thyroid) the patient did not receive Radioiodine ablation or treatment. Thyroglobulin and ant-thyroglobulin antibodies were undetectable three months after thyroidectomy. 

After 3 years of follow-up, the patient is well with her weight and has normal menstrual cycle. She is on levothyroxine 150 micgr in addition to carbonate calcium, vitamin D. The laboratory and radiology findings (neck sonography and brain MRI) are acceptable and the patient does not need to prednisolone or desmopressin.

**Figure 1:**
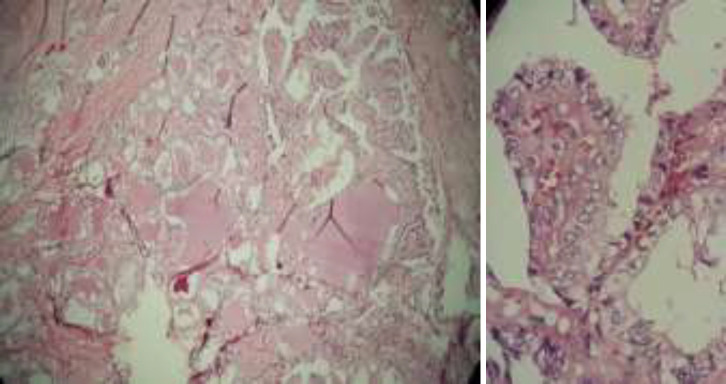
Patients pituitary adenoma months after thyroidectomy

**Figure 2:**
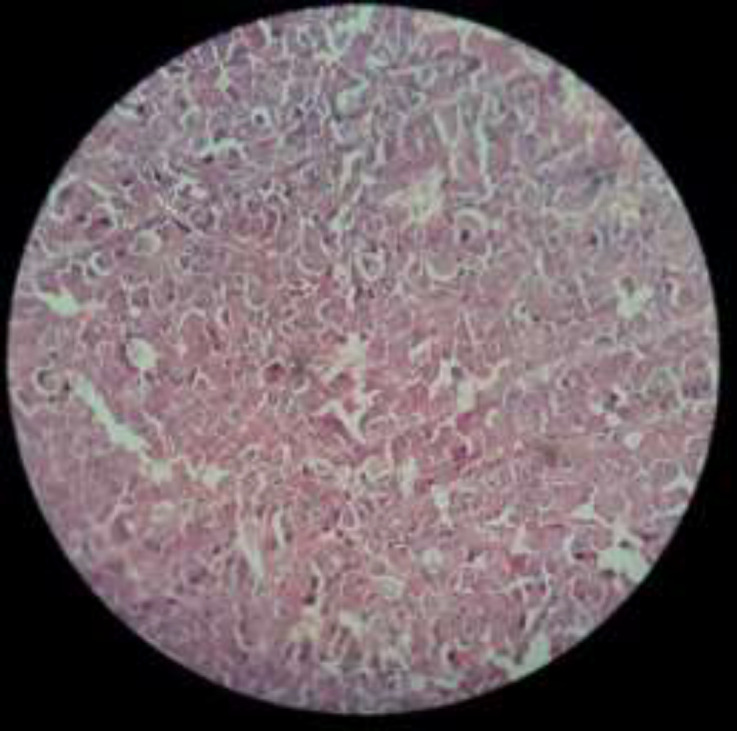
left lobe thyroid pathology

## Discussion

NTD is more frequent in patients with functional pituitary adenomas (2). About 8-16 percent of NTD are malignant. The thyroid PTC can be seen concurrent to some kind of pituitary tumors. Screening for PTC is recommended in patients with acromegaly because of increased risk of developing cancer in this group (3-13). Though an increased risk of NTD in patients with CD and prolactinoma has been reported in recent studies (3, 4, 7, 9, 10), but screening for PTC is not recommended yet (2-19). 

Synaptophysin is positive in normal anterior and posterior pituitary gland and all pituitary adenomas. Chromogranin A is characteristic for gonadotrope adenoma and is poorly expressed in GH adenomas. For the differentiation between GH secreting tumor and gonadotropes complementary tests should be done. The positivity of cells for ACTH and GH antibodies can be because of the cross reaction of GH Ab,s with ACTH adenoma or less probably ,the cross reaction of ACTH Ab, s with GH secreting adenoma (20).

In a recent study in Turkey, the prevalence of NTD was 36% in prolactinoma, 34% in CD and 60% in patients with acromegaly. Also, the frequency of PTC was higher in CD (11.4%) compared to patients with acromegaly (10.8%) (21). Sheng-Fong et al.reported a 58-year-old man in Taiwan that had advanced PTC and then presented with pituitary apoplexy .the final diagnosis was recurrent ACTH producing adenoma without cushingoid features (22). In another study in 1968 in the USA,2 cases of Cushing’s syndromes were reviewed which PTC was diagnosed after 2 and 5 years respectively (23). Kanazunorg et al. reported a 75- year- old woman with tumor in her pituitary, thyroid and left adrenal gland pituitary tumor was preclinical Cushing’s disease accompanied with PTC and adrenal incidentaloma (24).

The similar frequency of PTC seen among the different FPA groups may reflect their common etiopathogenic mechanisms, including cytokines or growth factors. Another possibility is that the genetic and epigenetic factors that cause pituitary adenoma development may also be responsible for PTC. May be a close follow-up of NTD in CD patients beneficial for the early diagnosis of PTC. Further studies on larger cohorts are needed to assess the prevalence of PTC, and to specify the related risk factors.

Our results suggested that patients with Cushing’s disease may be needed to a close follow up for NTD, as the papillary thyroid carcinoma was confirmed in this study. 

## References

[1] Hegedus L (2004). Clinical practice The thyroid nodule. N Eng J Med.

[2] Herrmann BL, Baumann H, Janssen OE (2004). Impact of disease activity on thyroid diseases in patients with acromegaly: basal evaluation and follow-up. Exp Clin Endocrinol Diabetes.

[3] Burman KD, Wartofsky L (2015). Clinical practice. thyroid nodules. N Eng J Med.

[4] Colao A, Pivonello R, Faggiano A (2000). Increased prevalence of thyroid autoimmunity in patients successfully treated for Cushing's disease. Clin Endocrinol.

[5] Dagdelen S, Cinar N, Erbas T (2014). Increased thyroid cancer risk in acromegaly. Pituitary.

[6] Gullu BE, Celik O, Gazioglu N, Kadioglu P (2010). Thyroid cancer is the most common cancer associated with acromegaly. Pituitary.

[7] Invitti C, Manfrini R, Romanini BM, Cavagnini F (1995). High prevalence of nodular thyroid disease in patients with Cushing's disease. Clin Endocrinol.

[8] Kaldrymidis D, Papadakis G, Tsakonas G (2016). High incidence of thyroid cancer among patients with acromegaly. J BUON.

[9] Sayki Arslan M, Sahin M, Topaloglu O (2013). Hyperprolactinaemia associated with increased thyroid volume and autoimmune thyroiditis in patients with prolactinoma. Clin Endocrinol.

[10] Tam AA, Kaya C, Aydin C, Ersoy R, Cakir B (2016). Differentiated thyroid cancer in patients with prolactinoma. Turkish J Med Sci.

[11] Terzolo M, Reimondo G, Berchialla P (2017). Acromegaly is associated with increased cancer risk: a survey in Italy. Endocrine-related cancer..

[12] Tirosh A, Shimon I (2017). Complications of acromegaly: thyroid and colon. Pituitary.

[13] Tita P, Ambrosio MR, Scollo C (2005). High prevalence of differentiated thyroid carcinoma in acromegaly. Clin Endocrinol.

[14] Reverter JL, Fajardo C, Resmini E (2014). Benign and malignant nodular thyroid disease in acromegaly Is a routine thyroid ultrasound evaluation advisable?. Plos One.

[15] Wolinski K, Czarnywojtek A, Ruchala M (2014). Risk of thyroid nodular disease and thyroid cancer in patients with acromegaly--meta-analysis and systematic review. Plos One.

[16] Dogan S, Atmaca A, Dagdelen S, Erbas B, Erbas T (2014). Evaluation of thyroid diseases and differentiated thyroid cancer in acromegalic patients. Endocrine.

[17] Uchoa HB, Lima GAB, Corrêa LL (2013). Prevalence of thyroid diseases in patients with acromegaly : experience of a Brazilian center. Arq Bras Endocrinol Metabol.

[18] Woliński K, Stangierski A, Gurgul E (2017). Thyroid lesions in patients with acromegaly — case-control study and update to the meta-analysis Thyroid lesions in patients with acromegaly— case-control study and update to the meta-analysis. Endokrynol Pol.

[19] dos Santos MC, Nascimento GC, Nascimento AG (2013). Thyroid cancer in patients with acromegaly: a case-control study. Pituitary.

[20] Goldblum JR, Lamps LW, McKenney JK, Myers JL (2017). Rosai and Ackerman's Surgical pathology e-book: Elsevier Health Sciences.

[21] Dogansen S, YenİDÜNya Yalin G, Tanrikulu S, Yarman S (2018). Nodular Thyroid Disease and Papillary Thyroid Carcinoma in Functional Pituitary Adenomas. Turk J Endocrinol Metab.

[22] Kuo SF, Chen JY, Chuang WY (2007). Concurrent papillary thyroid cancer with pituitary ACTH-secreting tumor. J Formosan Medl Assoc.

[23] Williams ED, Morales AM, Horn RC (1968). Thyroid carcinoma and Cushing's syndrome A report of two cases with a review of the common features of the "non-endocrine" tumours associated with Cushing's syndrome. J Clin Pathol.

[24] Kageyama K, Moriyama T, Sakihara S, Kawashima S, Suda T (2003). A case of preclinical Cushing's disease, accompanied with thyroid papillary carcinoma and adrenal incidentaloma. Endocrine J.

